# Supplementary material for: Hesperetin, a Promising Dietary Supplement for Preventing the Development of Calcific Aortic Valve Disease
Source: Antioxidants (Basel). 2022 Oct 24;11(11):2093. doi: 10.3390/antiox11112093 (PMC9687039; doi:10.3390/antiox11112093)
Supplement: Supplementary file 1 [file antioxidants-11-02093-s001.zip › antioxidants-1909578-supplementary.pdf]

Hesperetin, a promising dietary supplement for preventing the development of calcific aortic valve disease

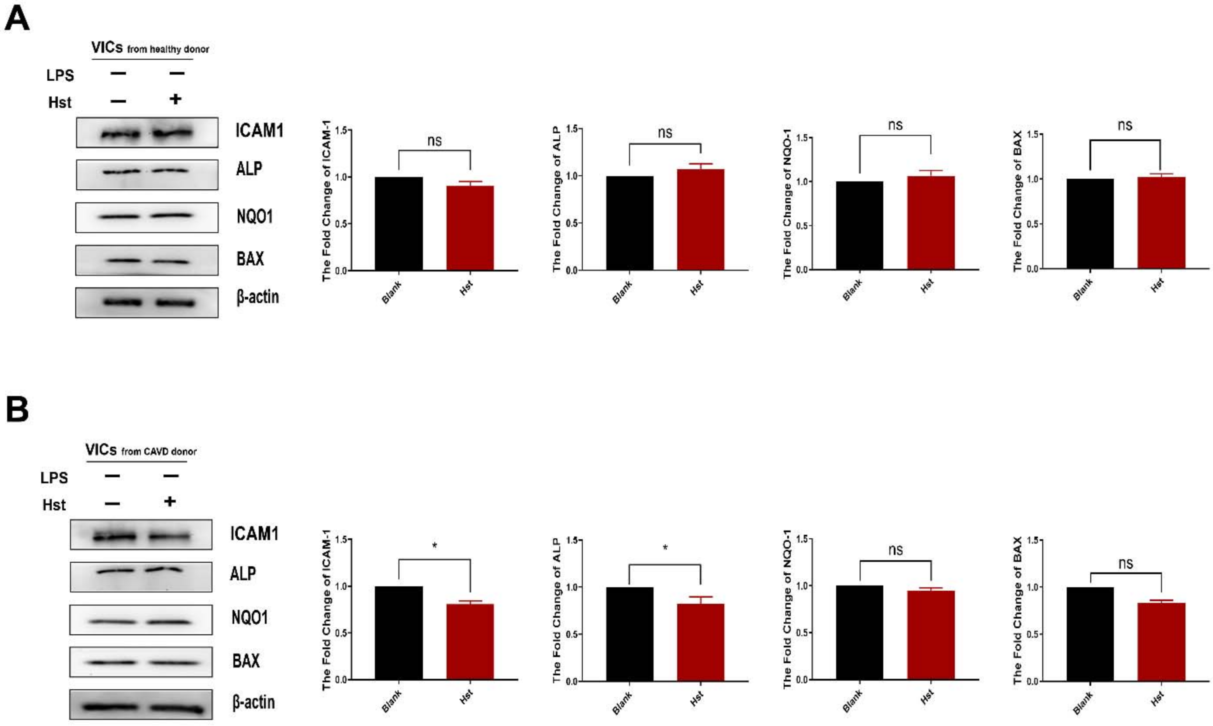

Supplemental Figure S1. The effect of hesperetin was examined in healthy and CAVD donors. (A) The expressions of ICAM-1, ALP, NQO-1 and BAX in healthy donors were detected by western blotting. n=3. (B) The expressions of ICAM-1, ALP, NQO-1 and BAX in CAVD donors were detected by western blotting. n= 3. * Represents $P < 0.05$, ** represents $P < 0.01$, *** represents $P < 0.001$, **** represents $P < 0.0001$ and n.s. represents $P > 0.05$.

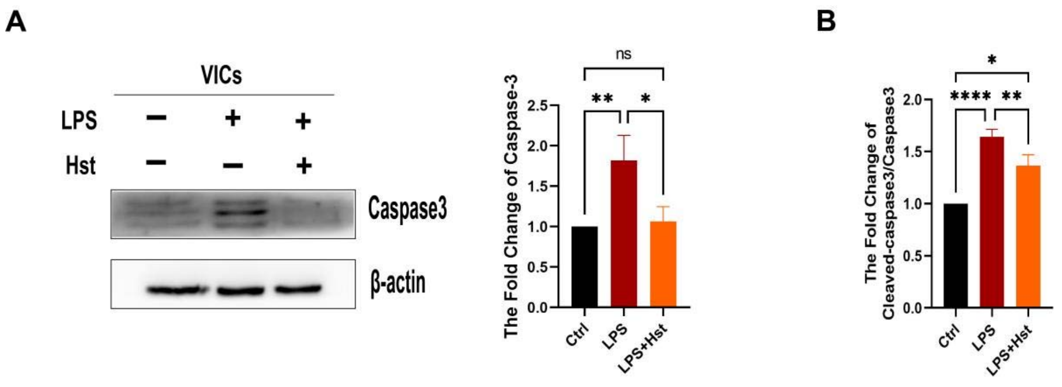

Supplemental Figure S2. Hesperetin inhibited the apoptosis of VICs under LPS stimulation. (A) The expressions of Caspase3 was detected by western blotting in each group. n= 3. (B) Calculated the ratio of Cleaved-caspase3 to caspase3. * Represents P < 0.05, ** represents P < 0.01, *** represents P < 0.001, **** represents P < 0.0001 and n.s. represents P > 0.05.

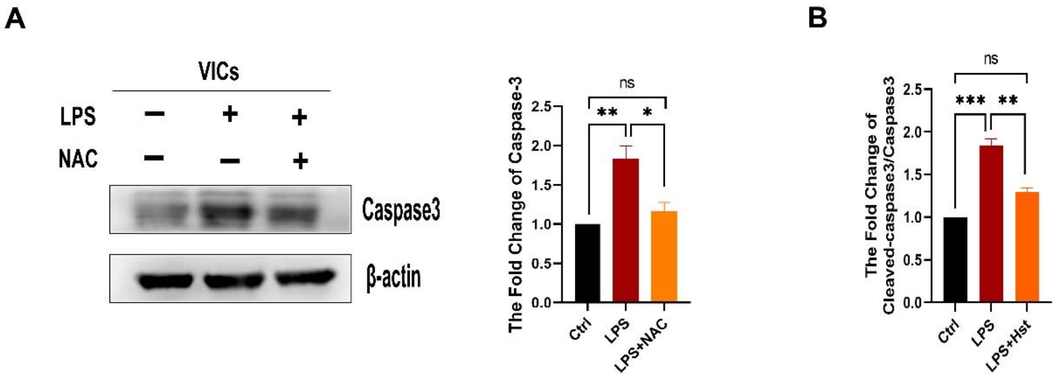

Supplemental Figure S3. NAC inhibited the apoptosis of VICs under LPS stimulation. (A) The expressions of Caspase3 was detected by western blotting in each group. n= 3. (B) Calculated the ratio of Cleaved-caspase3 to caspase3. * Represents P < 0.05, ** represents P < 0.01, *** represents P < 0.001, **** represents P < 0.0001 and n.s. represents P > 0.05.

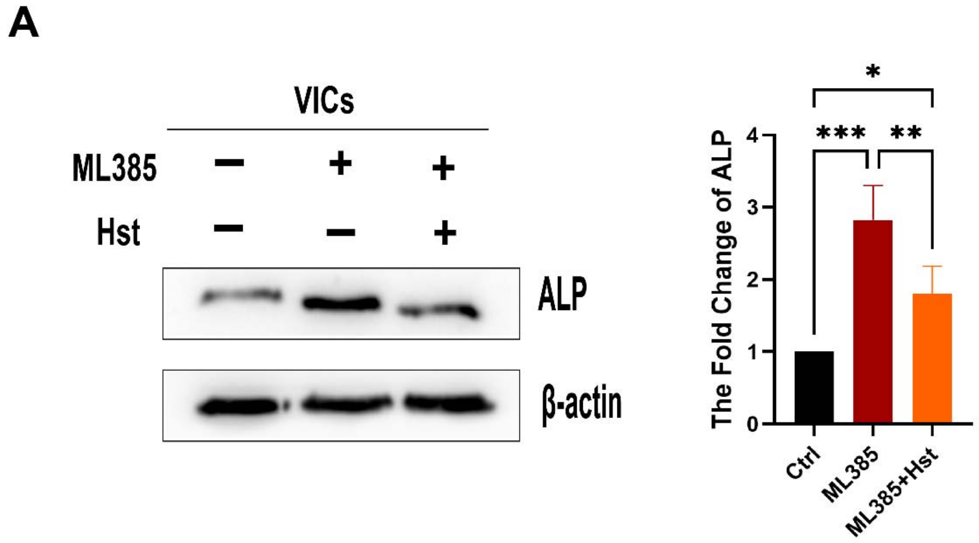

Supplemental Figure S4. Hesperetin inhibited the osteogenic response of VICs under ML385 stimulation. (A) The expressions of osteogenic protein (ALP) was detected by western blotting in each group. n= 3. * Represents $P < 0.05$, ** represents $P < 0.01$, *** represents $P < 0.001$, **** represents $P < 0.0001$ and n.s. represents $P > 0.05$.

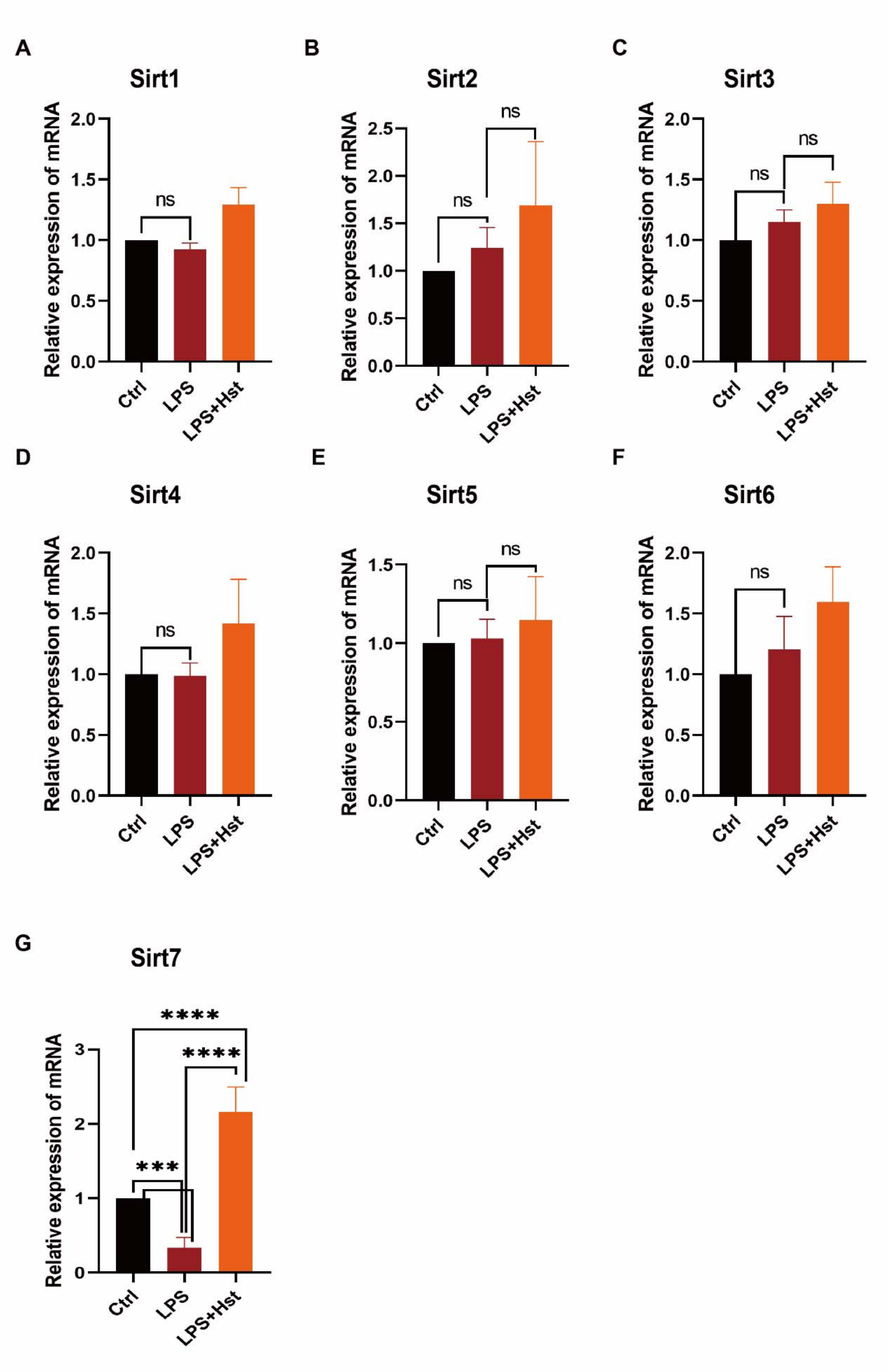

Supplemental Figure S5. Effects of hesperetin supplementation on sirtuin family

mRNA levels under LPS stimulation. (A-G) mRNA levels of sirtuin family members in human VICs under hesperetin supplementation for 24h. n= 5. * Represents P < 0.05, ** represents P < 0.01, *** represents P < 0.001, **** represents P < 0.0001 and n.s. represents P > 0.05.

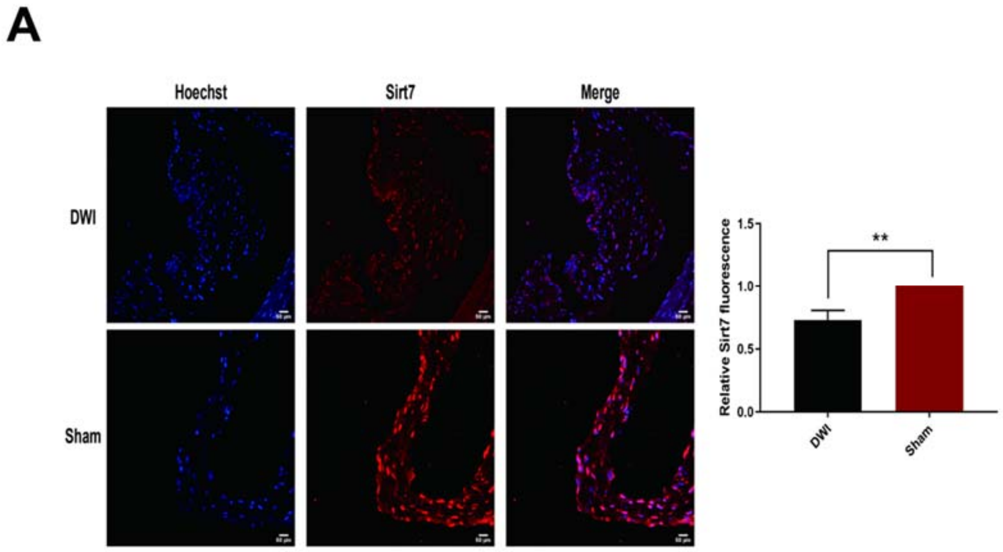

Supplemental Figure S6. Sirt7 expression was decreased in DWI mice. (A) The Sirt7 protein levels in mouse aortic valves were detected by immunofluorescence staning; Scale bar=50 μm, n= 5. * Represents P < 0.05, ** represents P < 0.01, *** represents P < 0.001, **** represents P < 0.0001 and n.s. represents P > 0.05.

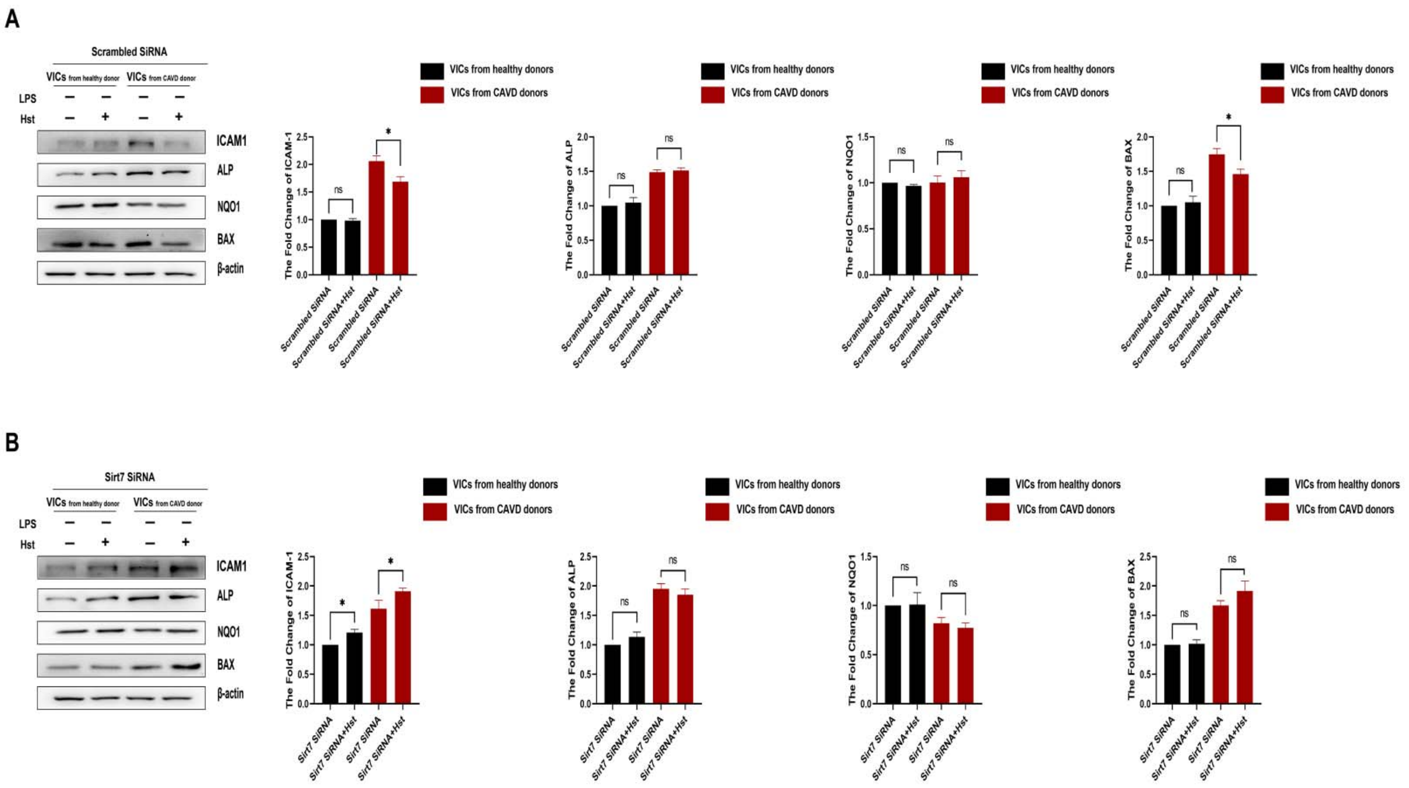

Supplemental Figure S7. The effect of hesperetin treatment alone was examined with Srcambled SIRNA or with Sirt7 SIRNA. (A) The expressions of ICAM-1, ALP, NQO-1 and BAX in Srcambled siRNA treatment were detected by western blotting. n= 3. (B) The expressions of ICAM-1, ALP, NQO-1 and BAX in Sirt7 SIRNA treatment were detected by western blotting. n= 3. * Represents $P < 0.05$, ** represents P <

0.01, *** represents P < 0.001, **** represents P < 0.0001 and n.s. represents P > 0.05.

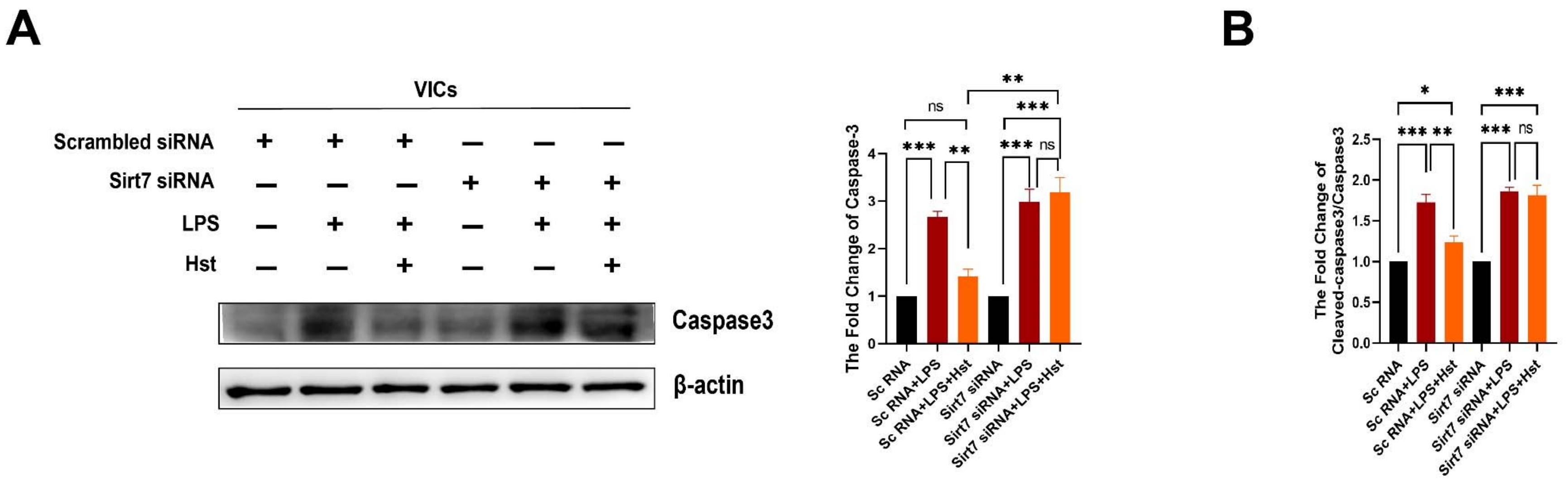

Supplemental Figure S8. The inhibitory effect of hesperetin on the apoptosis of VICs was abolished after knockdown of Sirt7.. (A) The expressions of Caspase3 was detected by western blotting in each group. n= 3. (B) Calculated the ratio of Cleaved-caspase3 to caspase3. * Represents P < 0.05, ** represents P < 0.01, *** represents P < 0.001, **** represents P < 0.0001 and n.s. represents P > 0.05.

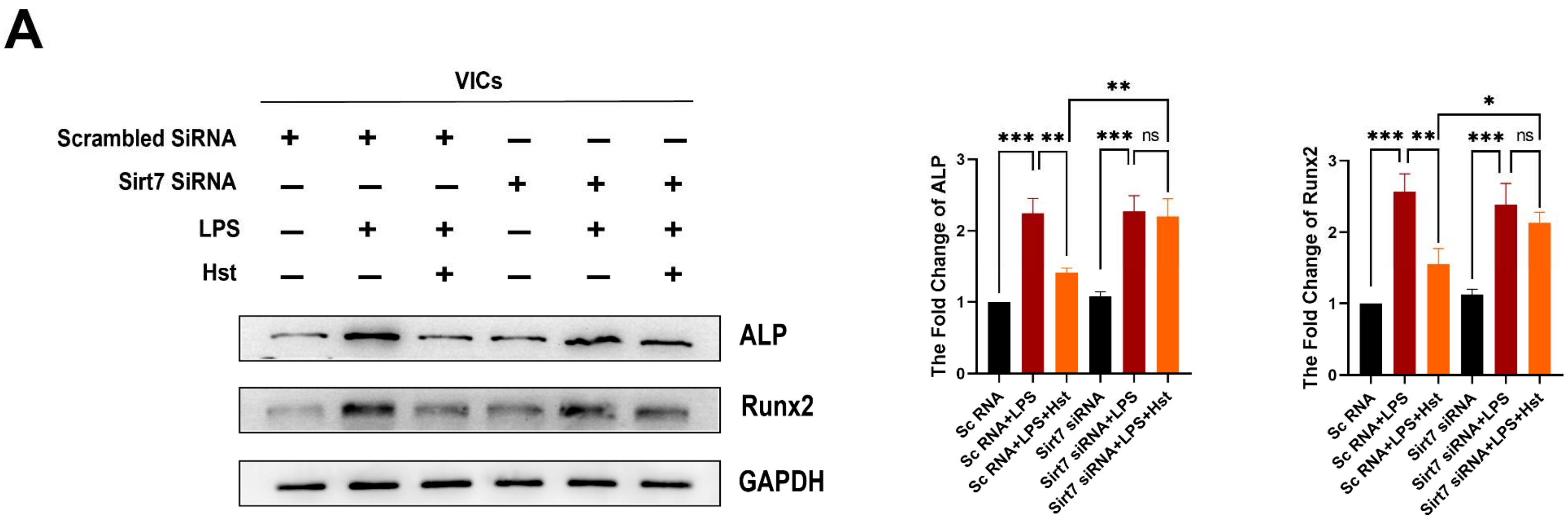

Supplemental Figure S9. The inhibitory effect of hesperetin on the osteogenic response of VICs was abolished after knockdown of Sirt7.

(A) The expressions of osteogenic proteins (ALP and Runx2) were detected by western blotting in each group. n= 3. * Represents P < 0.05, ** represents P < 0.01, *** represents P < 0.001, **** represents P < 0.0001 and n.s. represents P > 0.05.
